# Evaluating the Causal Roles of Matrix Metalloproteinases in Metabolic Syndrome: A Mendelian Randomization Study Highlighting MMP12 in Blood Pressure Regulation

**DOI:** 10.1155/genr/1665162

**Published:** 2026-08-30

**Authors:** Qiaohui Qian, Ming Fang, Zhaohua Gu, Yaming Yan, He Wen, Qian Sheng, Zeguo Shao, Xinming Li

**Affiliations:** ^1^ Department of Endocrinology, Zhoupu Hospital, Shanghai University of Medicine and Health Sciences, Shanghai 201318, China, sumhs.edu.cn; ^2^ Department of Cardiology, Longhua Hospital, Shanghai University of Traditional Chinese Medicine, Shanghai 200032, China, shutcm.edu.cn; ^3^ Zhoupu Community Health Service Center, Pudong New Area, Shanghai 201315, China; ^4^ Kangqiao Community Health Service Center, Pudong New Area, Shanghai 201318, China; ^5^ General Management Office, Zhoupu Hospital, Shanghai University of Medicine and Health Sciences, Shanghai 201318, China, sumhs.edu.cn; ^6^ College of Medical Instruments, Shanghai University of Medicine and Health Sciences, Shanghai 201318, China, sumhs.edu.cn; ^7^ Department of Cardiology, Zhoupu Hospital, Shanghai University of Medicine and Health Sciences, Shanghai 201318, China, sumhs.edu.cn

**Keywords:** essential hypertension, fasting blood glucose, matrix metalloproteinase, Mendelian randomization, metabolic syndrome

## Abstract

**Purpose:**

This study aims to investigate whether matrix metalloproteinases (MMPs) are associated with the risk of metabolic syndrome (MetS) and its components.

**Methods:**

We conducted a two‐sample Mendelian randomization (MR) study by using 9 types of MMPs as exposures and MetS and its components (waist circumference, essential hypertension, fasting blood glucose [FBG], high‐density lipoprotein [HDL] cholesterol, and triglycerides) as outcomes. The genome‐wide association study summary data of exposure and outcome were both obtained from publicly published research. Four MR analytical methods, including inverse variance weighted, MR‐Egger, weighted median, and weighted mode, were applied to analyze the causality between exposure and outcome, in which IVW was employed as the primary analytical method. MR‐Egger, Cochran’s Q, leave‐one‐out (LOO), and MR pleiotropy residual sum and outlier (MR‐PRESSO) were used to assess the reliability of the results of the MR analysis.

**Results:**

Genetically determined MMP12 showed significant negative associations to the risk of essential hypertension (OR = 0.996, 95% CI: 0.993–0.998, *p* = 0.001). Results that were analyzed with the MR‐Egger, weighted median, and weight mode were consistent with those analyzed with IVW. Although MMP12 may have a suggestive protective effect on FBG levels (OR = 0.993, 95% CI: 0.986–1, *p* = 0.049), this finding may be affected by potential horizontal pleiotropy and requires further validation. No causal associations were identified between other MMPs and the risk of MetS or its components.

**Conclusions:**

This MR analysis provides evidence of causal links between MMP12 and the risk of essential hypertension, indicating that MMP12 might inform early risk stratification for specific cardiometabolic components (e.g., essential hypertension), rather than serving as a diagnostic biomarker for the composite metabolic syndrome.

## 1. Introduction

MetS is a widespread disorder linked to the rising rates of obesity. MetS is characterized by a combination of metabolic issues, such as insulin resistance, central obesity, atherogenic dyslipidemia, and high blood pressure [1]. As a result of that, waist circumference, essential hypertension, fasting blood glucose (FBG), high‐density lipoprotein (HDL) cholesterol, and triglycerides are well‐recognized components of MetS. As the Western lifestyle spreads worldwide, MetS has become a global issue. In certain developing countries, urban populations often exhibit higher prevalence rates than those in Western nations [2, 3]. While certain factors, such as dietary habits and physical activity, have been clearly identified in the causation of MetS, the underlying pathophysiology of its occurrence remains unclear. Identifying reliable early indicators for risk stratification remains a clinical challenge, largely due to the multifactorial nature of the disease. Recently, the gut microbiome and inflammatory factors have been proven to play a role in regulating MetS [4–6]. In addition, the function of matrix metalloproteinases (MMPs) in regulating metabolism has been uncovered recently [7].

MMPs, as well as its inhibitors, TIMPs, are series of inflammatory factors that have been verified to function as inflammation and metabolism regulators in many diseases. MMPs are a family of over 20 zinc‐related enzymes, which participate in the restructuring of various elements within the extracellular matrix (ECM). MMPs are crucial in numerous physiological activities, including embryo implantation and organ development. Additionally, they contribute to tissue reorganization in pathological situations, such as inflammation, wound healing, and cancer cell invasion [8, 9]. In the context of cardiometabolic disorders, ECM remodeling driven by MMPs is increasingly recognized as a key theoretical framework linking adipose‐tissue dysfunction to systemic insulin resistance and vascular stiffness. Recently, a series of studies have revealed that several MMPs are associated with the risk of MetS. One study demonstrates that MMP9 and MMP3 are correlated with hypertension [10]. Another study focusing on type 2 diabetes mellitus has observed a significant increase of serum MMP12 level in type 2 diabetes patients when compared to healthy individuals [11]. These findings indicate MMPs may play a role in regulating the development of MetS. However, these findings are largely derived from cross‐sectional or cohort studies and are therefore susceptible to residual confounding and reverse causality, making it difficult to determine whether altered MMP levels are a cause or a consequence of cardiometabolic disorders.

Mendelian randomization (MR) analysis, a technique for assessing the causal connections between exposures and outcomes, could be a powerful tool for causal analysis [12]. By utilizing genetic variants as instrumental variables (IVs), MR analysis helps infer causality with reduced confounding issues [13]. Notably, despite growing observational evidence implicating MMP12 in hypertension, dysglycemia, and vascular disease, there is a lack of systematic MR studies evaluating whether these associations reflect true causal effects.

In this study, we performed a two‐sample MR analysis to systematically investigate the potential causal effects of nine MMPs on MetS and its key components, including blood pressure and fasting glucose. By leveraging large‐scale genome‐wide association study (GWAS) summary statistics and multiple complementary MR methods, this study aims to compare the causal relevance of different MMP subtypes, clarify whether previously reported associations—particularly for MMP12—are consistent with a causal interpretation, and distinguish component‐specific effects from the composite MetS phenotype.

## 2. Material and Methods

### 2.1. Study Design

**FIGURE 1:**
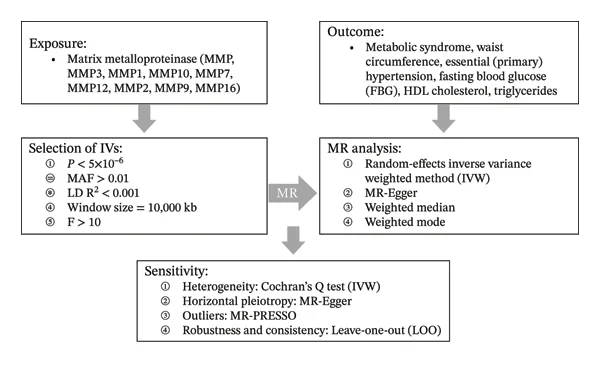
Schematic illustration of the two‐sample MR study. MMPs, matrix metalloproteinase; IVW, inverse variance weighted; MR, Mendelian randomization; MAF, minor allele frequency; LD, linkage disequilibrium; MR‐PRESSO, MR pleiotropy residual sum and outlier.

In this study, we conducted two‐sample MR analyses to assess the causal associations between MMPs and MetS. As illustrated in Figure 1, we first identified 9 types of MMPs as exposure, while MetS along with 5 components were outcomes. These components included waist circumference, essential hypertension, FBG, HDL cholesterol, and triglycerides. Upon acquiring the GWAS data, we selected SNPs that met the following three criteria as IVs: (1) These SNPs had a direct association with the exposure; (2) there was no genetic correlation between these SNPs and the outcome; and (3) there was no association between the selected SNPs and potential confounders [14]. These IVs were used for MR and sensitivity analyses. The entire analytical procedure followed the “Strengthening the Reporting of Observational Studies in Epidemiology using Mendelian Randomization” (STROBE‐MR) guidelines [15].

### 2.2. Data Sources

The data sources and information of the GWAS data of exposures and outcomes are listed in Supporting Table 1. Briefly, for the outcome, GWAS data for MetS were obtained from data in the UK Biobank in 291,107 European ancestry individuals [16]. For waist circumference, the data originated from the UK Biobank dataset with up to 407,746 British ancestry individuals [17]. Similarly, GWAS data for essential hypertension were gathered from IEU Open GWAS Project in 54,358 cases and 408,652 control European ancestry individuals. Meanwhile, the GWAS data for FBG came from GWAS comprising up to 200,622 individuals [18]. GWAS data of HDL cholesterol and triglycerides were achieved from a published study that examined 94,595 European ancestry individuals [19]. For the exposure, GWAS data of MMP were obtained from genome‐ and epigenome‐wide association studies (GWAS/EWAS) with 982 Scottish ancestry individuals [20]. GWAS data of MMP3, MMP1, MMP10, MMP7, and MMP12 were gathered from a genomic and drug target evaluation among 21,758 European ancestry individuals [21]. Meanwhile, GWAS data of MMP2 and MMP9 originated from a large‐scale proteogenomic study among Icelandic ancestry individuals [22]. GWAS data of MMP16 came from genomic atlas of the human plasma proteome in 3301 European ancestry individuals [23]. All GWAS data from the included articles were approved by corresponding ethics committees, and participants provided written informed consent.

### 2.3. Selection of IVs

To select IVs for MR analysis from the GWAS data, we applied several criteria: (1) Due to an insufficient number of SNPs satisfying the threshold of *p* < 5 × 10^−8^, in this study, the threshold for IV selection was adopted to *p* < 5 × 10^−6^, a standard that was utilized in a previous study [24]; (2) the minor allele frequency (MAF) for the selected SNP had to be greater than 0.01 [25]; (3) the SNPs with linkage disequilibrium (LD) effect were excluded by ensuring an *R* ^2^ < 0.001 within a 10,000 kb window [26, 27]; (4) if an IV was missing in the outcome summary, it was replaced with a proxy one with high LD (*R* ^2^ > 0.8) [28]; and (5) to prevent weak instrument bias, F‐statistic was calculated using the formula: F = *R* ^2^ × (N−2)/(1‐*R* ^2^), where *R* ^2^ statistic represented the variance in the exposure explained by the genetic variants. The SNPs included as IVs were required to reach an F‐statistic greater than 10 [29].

### 2.4. MR Analysis

To explore the causal relationships between MMPs and MetS, this study employed a two‐sample MR analysis utilizing four distinct MR techniques: inverse variance weighted (IVW), MR‐Egger, weighted median, and weighted mode. The main method, IVW, calculated the weighted average of effect sizes using the inverse variance of each SNP as a weight, which enabled the estimation of odds ratios (OR) and 95% confidence intervals (CI) [30]. The techniques, MR‐Egger, weighted median, and weighted mode, supported the IVW findings [31–34]. Specifically, MR‐Egger adjusted for pleiotropic bias to provide accurate causal effect estimates through its intercept. The weighted median approach assumed that at least half of the IVs were valid to determine causal relationships between exposure and outcome. Furthermore, the weighted mode method, which identifies the mode of the empirical density function of the ratio estimates, delivered reliable results even when horizontal pleiotropy was present [35]. The analyses were performed using R software (Version 4.0.5) and the “TwoSampleMR” package, with results displayed in forest plots and scatter plots.

### 2.5. Sensitivity Analysis

In the sensitivity analysis, we used four different methods to evaluate potential horizontal pleiotropy and heterogeneity. To assess heterogeneity among the IVs in each MR analysis, we performed the IVW Cochran’s Q test, where a *p*‐value greater than 0.05 indicated low heterogeneity and minimal effect on the IVW results [36]. To investigate horizontal pleiotropy, the MR‐Egger method was applied; a nonsignificant intercept or one close to zero suggested low pleiotropy [37]. Furthermore, to identify and remove outliers, the MR‐PRESSO test was used, excluding SNPs with a *p*‐value of less than 0.05 [38]. Finally, a leave‐one‐out (LOO) analysis was carried out to test the robustness of the findings and to determine whether any single IV was disproportionately affecting the causal relationship between the exposure and outcome [39].

## 3. Results

### 3.1. Selection of IVs

Following the predefined screening criteria, we identified a total of 178 SNPs associated with the nine MMP traits as IVs for MR analysis (Supporting Table 2). The mean F‐statistics for the nine exposures ranged from 22.39 to 127.34, all well above the conventional threshold of 10, indicating that weak instrument bias was unlikely to affect the causal estimates. Detailed SNP counts and F‐statistics for each MMP trait are provided in Supporting Table 3.

### 3.2. Causal Effects of MMPs on MetS

**FIGURE 2:**
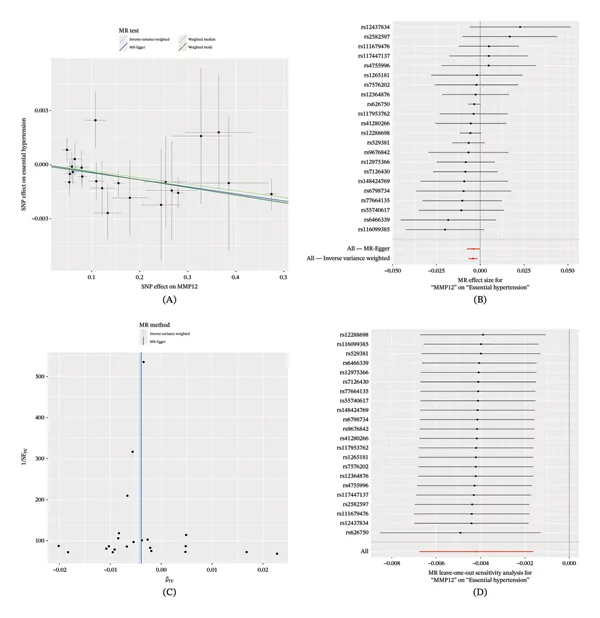
MR analysis and sensitivity analysis of causal relationship between MMP12 and essential hypertension. (A) Scatter plot of the association between MMP12 and essential hypertension. The 4 MR methods applied in this study were all depicted, including IVW, MR‐Egger, weight mode, and weight median MR methods. (B) Forest plot that showed the MR estimate and 95% CI values for each included SNP, as well as the IVW and MR‐Egger results. (C) Funnel plot to detect whether the observed association between MMP12 and essential hypertension was along with obvious heterogeneity. (D) Leave‐one‐out analysis to evaluate whether any single instrumental variable was driving the causal effect of MMP12 on essential hypertension.

To investigate the causal relationship between 9 types of MMPs and MetS as well as its components, we carried out a series of univariable two‐sample MR analyses, examining one MMP and one MetS component in each analysis. The results of the MR analyses were presented in forest plots and are summarized in Table 1. Notably, genetically determined MMP12 showed a significant negative association with the risk of essential hypertension (OR = 0.996, 95% CI: 0.993–0.998, *p* = 0.001). These results were consistent across different models, including weighted median (OR = 0.996, 95% CI: 0.993–1, *p* = 0.034) and weighted mode (OR = 0.996, 95% CI: 0.993–0.999, *p* = 0.011) (Table 1). Although the results of MR‐Egger did not show a significant association between MMP12 and essential hypertension (OR = 0.996, 95% CI: 0.992–1, *p* = 0.058), the scatter plot of effect sizes of IVs indicated similar estimated results across four different methods (Figure 2A). Moreover, the forest plot consistently highlighted a negative relationship between MMP12 and the risk of essential hypertension (Figure 2B). In the sensitivity analysis, IVW Cochran’s Q test indicated no significant heterogeneity (*p* > 0.05). Additionally, the MR‐Egger intercept regression showed no evidence of pleiotropy (*p* > 0.05) (Table 2), and the MR‐PRESSO test further confirmed the insignificant horizontal pleiotropy, as summarized in Table 3. Moreover, the symmetry of the funnel plot suggested negligible biases (Figure 2C), while the LOO analysis indicated that excluding any SNPs would barely affect the causal estimates (Figure 2D).

**TABLE 1:** Associations between MMPs and the risk of metabolic syndrome and its components by 4 independent Mendelian randomization methods.

Exposure	Outcome	N.SNPs	Methods	OR (95% CI)	*p*
MMP3	Metabolic syndrome	18	IVW	0.983 (0.939–1.030)	0.473
			MR‐Egger	0.990 (0.921–1.065)	0.795
			Weighted median	0.989 (0.956–1.023)	0.522
			Weighted mode	0.989 (0.958–1.021)	0.504

MMP3	Waist circumference	20	IVW	0.993 (0.982–1.005)	0.259
			MR‐Egger	1.008 (0.992–1.024)	0.359
			Weighted median	0.999 (0.989–1.008)	0.782
			Weighted mode	0.997 (0.988–1.006)	0.567

MMP3	Essential hypertension	19	IVW	0.997 (0.995–1.000)	0.056
			MR‐Egger	0.998 (0.994–1.002)	0.358
			Weighted median	0.998 (0.994–1.001)	0.195
			Weighted mode	0.998 (0.995–1.001)	0.233

MMP3	FBG	21	IVW	1.005 (0.994–1.015)	0.385
			MR‐Egger	1.017 (1.002–1.032)	0.034
			Weighted median	1.007 (0.998–1.017)	0.115
			Weighted mode	1.008 (0.999–1.017)	0.108

MMP3	HDL cholesterol	6	IVW	1.038 (0.972–1.108)	0.268
			MR‐Egger	0.924 (0.816–1.046)	0.28
			Weighted median	1.004 (0.945–1.066)	0.894
			Weighted mode	1.005 (0.945–1.069)	0.878

MMP3	Triglycerides	6	IVW	1.038 (0.972–1.108)	0.268
			MR‐Egger	0.924 (0.816–1.046)	0.28
			Weighted median	1.004 (0.945–1.066)	0.894
			Weighted mode	1.005 (0.945–1.069)	0.878

MMP	Metabolic syndrome	5	IVW	1.002 (0.980–1.025)	0.849
			MR‐Egger	0.950 (0.860–1.050)	0.389
			Weighted median	0.997 (0.971–1.024)	0.826
			Weighted mode	0.997 (0.966–1.029)	0.862

MMP	Waist circumference	5	IVW	0.999 (0.992–1.006)	0.72
			MR‐Egger	1.017 (0.989–1.046)	0.327
			Weighted median	1.001 (0.994–1.009)	0.723
			Weighted mode	1.002 (0.994–1.011)	0.606

MMP	Essential hypertension	5	IVW	1.000 (0.997–1.002)	0.677
			MR‐Egger	0.999 (0.989–1.009)	0.881
			Weighted median	1.000 (0.997–1.002)	0.823
			Weighted mode	1.000 (0.997–1.003)	0.867

MMP	FBG	5	IVW	0.997 (0.990–1.004)	0.362
			MR‐Egger	0.971 (0.943–0.999)	0.136
			Weighted median	0.997 (0.988–1.005)	0.47
			Weighted mode	0.990 (0.981–0.999)	0.101

MMP	HDL cholesterol	3	IVW	0.976 (0.951–1.001)	0.064
			MR‐Egger	0.918 (0.813–1.036)	0.397
			Weighted median	0.982 (0.952–1.014)	0.271
			Weighted mode	0.984 (0.951–1.018)	0.449

MMP	Triglycerides	3	IVW	0.988 (0.964–1.013)	0.343
			MR‐Egger	1.031 (0.920–1.156)	0.689
			Weighted median	0.989 (0.958–1.020)	0.484
			Weighted mode	0.990 (0.954–1.027)	0.645

MMP1	Metabolic syndrome	24	IVW	1.013 (0.982–1.045)	0.424
			MR‐Egger	1.015 (0.962–1.071)	0.602
			Weighted median	1.013 (0.978–1.049)	0.472
			Weighted mode	1.015 (0.980–1.051)	0.421

MMP1	Waist circumference	21	IVW	0.999 (0.988–1.010)	0.845
			MR‐Egger	1.002 (0.984–1.020)	0.833
			Weighted median	1.001 (0.991–1.011)	0.85
			Weighted mode	1.001 (0.991–1.011)	0.846

MMP1	Essential hypertension	22	IVW	1.001 (0.997–1.004)	0.713
			MR‐Egger	1.003 (0.997–1.009)	0.333
			Weighted median	1.002 (0.999–1.006)	0.201
			Weighted mode	1.002 (0.998–1.005)	0.304

MMP1	FBG	25	IVW	0.995 (0.988–1.003)	0.222
			MR‐Egger	1.000 (0.988–1.012)	0.951
			Weighted median	0.996 (0.987–1.005)	0.393
			Weighted mode	0.997 (0.988–1.006)	0.476

MMP1	HDL cholesterol	3	IVW	0.974 (0.875–1.083)	0.627
			MR‐Egger	0.794 (0.533–1.181)	0.458
			Weighted median	0.953 (0.902–1.006)	0.081
			Weighted mode	0.948 (0.899–1.001)	0.194

MMP1	Triglycerides	3	IVW	1.010 (0.962–1.060)	0.694
			MR‐Egger	1.035 (0.859–1.247)	0.778
			Weighted median	1.011 (0.960–1.064)	0.686
			Weighted mode	1.012 (0.954–1.073)	0.725

MMP10	Metabolic syndrome	21	IVW	0.976 (0.943–1.010)	0.167
			MR‐Egger	0.997 (0.952–1.044)	0.895
			Weighted median	0.977 (0.944–1.010)	0.175
			Weighted mode	0.982 (0.950–1.015)	0.302

MMP10	Waist circumference	18	IVW	1.000 (0.980–1.021)	0.976
			MR‐Egger	0.987 (0.961–1.014)	0.364
			Weighted median	0.992 (0.983–1.002)	0.121
			Weighted mode	0.992 (0.982–1.002)	0.132

MMP10	Essential hypertension	21	IVW	1.002 (0.998–1.006)	0.342
			MR‐Egger	1.001 (0.995–1.007)	0.679
			Weighted median	1 (0.997–1.004)	0.827
			Weighted mode	1 (0.997–1.004)	0.788

MMP10	FBG	22	IVW	1.007 (0.991–1.022)	0.409
			MR‐Egger	0.993 (0.972–1.013)	0.487
			Weighted median	1 (0.988–1.012)	0.995
			Weighted mode	0.999 (0.988–1.011)	0.9

MMP10	HDL cholesterol	5	IVW	0.963 (0.918–1.009)	0.11
			MR‐Egger	0.966 (0.881–1.059)	0.517
			Weighted median	0.971 (0.931–1.013)	0.175
			Weighted mode	0.946 (0.904–0.989)	0.072

MMP10	Triglycerides	5	IVW	0.968 (0.9–1.041)	0.378
			MR‐Egger	1.007 (0.881–1.15)	0.929
			Weighted median	0.998 (0.955–1.042)	0.918
			Weighted mode	0.994 (0.951–1.04)	0.813

MMP7	Metabolic syndrome	21	IVW	1.006 (0.965–1.050)	0.763
			MR‐Egger	1.024 (0.959–1.092)	0.491
			Weighted median	1.022 (0.976–1.069)	0.360
			Weighted mode	1.019 (0.975–1.064)	0.413

MMP7	Waist circumference	20	IVW	1.006 (0.990–1.022)	0.496
			MR‐Egger	1.007 (0.981–1.033)	0.614
			Weighted median	1.006 (0.993–1.018)	0.368
			Weighted mode	1.006 (0.993–1.019)	0.386

MMP7	Essential hypertension	20	IVW	1.000 (0.995–1.004)	0.888
			MR‐Egger	0.997 (0.990–1.004)	0.477
			Weighted median	0.998 (0.994–1.003)	0.451
			Weighted mode	0.997 (0.993–1.002)	0.291

MMP7	FBG	23	IVW	1.005 (0.995–1.015)	0.312
			MR‐Egger	1.008 (0.992–1.024)	0.342
			Weighted median	1.004 (0.990–1.017)	0.607
			Weighted mode	1.004 (0.990–1.019)	0.557

MMP7	HDL cholesterol	7	IVW	0.997 (0.959–1.037)	0.888
			MR‐Egger	1.009 (0.955–1.066)	0.766
			Weighted median	1.004 (0.960–1.050)	0.855
			Weighted mode	1.003 (0.954–1.055)	0.903

MMP7	Triglycerides	7	IVW	0.963 (0.927–1.000)	0.051
			MR‐Egger	0.980 (0.929–1.033)	0.485
			Weighted median	0.975 (0.935–1.018)	0.248
			Weighted mode	0.975 (0.935–1.018)	0.290

MMP12	MetS	20	IVW	1.007 (0.978–1.038)	0.624
			MR‐Egger	1.013 (0.969–1.06)	0.574
			Weighted median	1.011 (0.978–1.046)	0.518
			Weighted mode	1.017 (0.985–1.05)	0.309

MMP12	Waist circumference	22	IVW	0.999 (0.99–1.007)	0.757
			MR‐Egger	0.998 (0.985–1.011)	0.725
			Weighted median	0.999 (0.99–1.009)	0.895
			Weighted mode	0.999 (0.99–1.009)	0.882

MMP12	Essential hypertension	22	IVW	0.996 (0.993–0.998)	0.001
			MR‐Egger	0.996 (0.992–1)	0.058
			Weighted median	0.996 (0.993–1)	0.034
			Weighted mode	0.996 (0.993–0.999)	0.011

MMP12	FBG	25	IVW	0.993 (0.986–1)	0.049
			MR‐Egger	0.984 (0.973–0.994)	0.005
			Weighted median	0.989 (0.98–0.998)	0.015
			Weighted mode	0.988 (0.98–0.996)	0.009

MMP12	HDL cholesterol	6	IVW	0.986 (0.964–1.009)	0.243
			MR‐Egger	0.973 (0.942–1.005)	0.174
			Weighted median	0.981 (0.958–1.005)	0.122
			Weighted mode	0.981 (0.958–1.005)	0.182

MMP12	Triglycerides	6	IVW	1.019 (0.996–1.042)	0.102
			MR‐Egger	1.021 (0.989–1.054)	0.262
			Weighted median	1.019 (0.996–1.043)	0.100
			Weighted mode	1.019 (0.995–1.044)	0.176

MMP2	MetS	18	IVW	0.981 (0.9297–1.0351)	0.4842
			MR‐Egger	1.0762 (0.9383–1.2343)	0.3096
			Weighted median	0.9813 (0.9315–1.0337)	0.4762
			Weighted mode	0.9431 (0.8597–1.0345)	0.2313

MMP2	Waist circumference	20	IVW	1.0056 (0.9908–1.0206)	0.4592
			MR‐Egger	1.0137 (0.9761–1.0528)	0.489
			Weighted median	1.0005 (0.986–1.0152)	0.949
			Weighted mode	1.0176 (0.9898–1.0462)	0.2328

MMP2	Essential hypertension	19	IVW	0.9989 (0.9931–1.0048)	0.7167
			MR‐Egger	0.9962 (0.9808–1.0117)	0.6335
			Weighted median	0.999 (0.9932–1.0048)	0.7236
			Weighted mode	0.9991 (0.9891–1.0092)	0.8656

MMP2	FBG	20	IVW	1.0138 (0.9848–1.0436)	0.3551
			MR‐Egger	0.9874 (0.9146–1.0661)	0.7501
			Weighted median	0.9963 (0.9826–1.0101)	0.5941
			Weighted mode	0.993 (0.97–1.0166)	0.5658

MMP2	HDL cholesterol	6	IVW	1.0366 (0.9697–1.108)	0.2909
			MR‐Egger	0.9838 (0.7323–1.3216)	0.9188
			Weighted median	1.018 (0.9558–1.0843)	0.5785
			Weighted mode	0.9811 (0.8647–1.1133)	0.7797

MMP2	Triglycerides	6	IVW	0.8366 (0.6558–1.0671)	0.1507
			MR‐Egger	0.8292 (0.273–2.519)	0.7577
			Weighted median	0.9768 (0.9164–1.0412)	0.4713
			Weighted mode	0.9815 (0.9151–1.0528)	0.6242

MMP9	MetS	19	IVW	1.0081 (0.9723–1.0453)	0.6615
			MR‐Egger	1.0181 (0.9429–1.0994)	0.6523
			Weighted median	1.0216 (0.976–1.0693)	0.3598
			Weighted mode	1.0245 (0.9672–1.0852)	0.4208

MMP9	Waist circumference	20	IVW	1.0015 (0.9898–1.0132)	0.8075
			MR‐Egger	0.9865 (0.9637–1.0097)	0.2671
			Weighted median	0.9995 (0.9872–1.0119)	0.9315
			Weighted mode	0.9998 (0.9837–1.0161)	0.9777

MMP9	Essential hypertension	19	IVW	1.0019 (0.9988–1.0049)	0.229
			MR‐Egger	1.0026 (0.9963–1.009)	0.4319
			Weighted median	1.003 (0.9986–1.0074)	0.1873
			Weighted mode	1.0037 (0.9986–1.0088)	0.1763

MMP9	FBG	20	IVW	1.0046 (0.996–1.0133)	0.2938
			MR‐Egger	0.9946 (0.9784–1.011)	0.5237
			Weighted median	1.0058 (0.993–1.0188)	0.3744
			Weighted mode	1.0056 (0.9918–1.0196)	0.4391

MMP9	HDL cholesterol	6	IVW	0.9813 (0.9422–1.022)	0.3618
			MR‐Egger	1.0061 (0.9348–1.0828)	0.8797
			Weighted median	0.9959 (0.9582–1.035)	0.8339
			Weighted mode	0.999 (0.9577–1.0421)	0.9652

MMP9	Triglycerides	6	IVW	1.0119 (0.9801–1.0448)	0.4671
			MR‐Egger	1.0251 (0.9651–1.0887)	0.466
			Weighted median	1.0177 (0.9802–1.0567)	0.3599
			Weighted mode	1.0202 (0.9805–1.0615)	0.3677

MMP16	MetS	10	IVW	1.016 (0.971–1.064)	0.488
			MR‐Egger	1.023 (0.892–1.173)	0.749
			Weighted median	1.026 (0.978–1.076)	0.291
			Weighted mode	1.032 (0.958–1.111)	0.434

MMP16	Waist circumference	10	IVW	1.008 (0.997–1.019)	0.173
			MR‐Egger	1.004 (0.972–1.038)	0.799
			Weighted median	1.001 (0.987–1.014)	0.916
			Weighted mode	0.997 (0.974–1.020)	0.79

MMP16	Essential hypertension	10	IVW	0.999 (0.995–1.003)	0.771
			MR‐Egger	1.006 (0.994–1.017)	0.363
			Weighted median	1.000 (0.995–1.005)	0.892
			Weighted mode	1.005 (0.996–1.013)	0.34

MMP16	FBG	13	IVW	1.002 (0.993–1.012)	0.653
			MR‐Egger	0.990 (0.965–1.016)	0.468
			Weighted median	1.002 (0.989–1.015)	0.768
			Weighted mode	0.998 (0.978–1.018)	0.828

MMP16	Waist circumference	10	IVW	1.008 (0.997–1.019)	0.173
			MR‐Egger	1.004 (0.972–1.038)	0.799
			Weighted median	1.001 (0.987–1.014)	0.916
			Weighted mode	0.997 (0.974–1.020)	0.790

MMP16	HDL cholesterol	5	IVW	0.994 (0.948–1.043)	0.807
			MR‐Egger	1.023 (0.865–1.211)	0.807
			Weighted median	0.980 (0.931–1.030)	0.422
			Weighted mode	0.971 (0.909–1.037)	0.425

MMP16	Triglycerides	5	IVW	0.981 (0.946–1.017)	0.297
			MR‐Egger	0.923 (0.826–1.032)	0.254
			Weighted median	0.977 (0.933–1.022)	0.308
			Weighted mode	0.976 (0.910–1.046)	0.53

*Note:* N. SNPs, number of SNPs used in MR analysis.

Abbreviations: CI, confidence interval; FBG, fasting blood glucose; HDL, high‐density lipoprotein; IVW, inverse variance weighted; OR, odds ratio.

**TABLE 2:** Heterogeneity and pleiotropy test of IVs.

Exposure	Outcome	Heterogeneity		Pleiotropy	
		Q‐statistic (IVW)	*p*‐value	MR‐Egger intercept	*p*‐value
MMP3	Metabolic syndrome	49.385	5.259261e−05	−0.001	0.800
	Waist circumference	43.465	0.001	−0.003	0.031
	Essential hypertension	11.880	0.853	−0.0001	0.678
	FBG	40.026	0.005	−0.002	0.040
	HDL cholesterol	7.465	0.188	0.011	0.112
	Triglycerides	13.630	0.018	−0.010	0.296

MMP	Metabolic syndrome	2.194	0.700	0.017	0.362
	Waist circumference	4.575	0.334	−0.006	0.288
	Essential hypertension	0.371	0.985	0.0001	0.947
	FBG	4.842	0.304	0.008	0.161
	HDL cholesterol	1.042	0.594	0.017	0.494
	Triglycerides	1.148	0.563	−0.012	0.588

MMP1	Metabolic syndrome	32.039	0.099	−0.0003	0.936
	Waist circumference	36.080	0.015	−0.0005	0.683
	Essential hypertension	32.014	0.058	−0.0004	0.345
	FBG	16.464	0.871	−0.0007	0.398
	HDL cholesterol	9.174	0.010	0.020	0.485
	Triglycerides	0.080	0.961	−0.002	0.832

MMP10	Metabolic syndrome	34.909	0.021	−0.005	0.207
	Waist circumference	128.641	3.42199804089505E−19	0.003	0.170
	Essential hypertension	48.775	0.0003	0.0001	0.714
	FBG	73.175	0.0000001	0.003	0.073
	HDL cholesterol	6.731	0.151	−0.001	0.927
	Triglycerides	17.186	0.002	−0.007	0.527

MMP7	Metabolic syndrome	32.238	0.041	−0.003	0.510
	Waist circumference	53.387	0.00004	−0.0001	0.917
	Essential hypertension	35.109	0.014	0.0004	0.408
	FBG	21.004	0.520	−0.0004	0.677
	HDL cholesterol	2.528	0.865	−0.002	0.579
	Triglycerides	4.903	0.556	−0.003	0.397

MMP12	Metabolic syndrome	25.197	0.154	−0.001	0.739
	Waist circumference	29.634	0.100	0.0002	0.836
	Essential hypertension	12.863	0.913	−0.00007	0.840
	FBG	18.646	0.771	0.002	0.022
	HDL cholesterol	3.803	0.578	0.004	0.308
	Triglycerides	1.631	0.897	−0.001	0.849

MMP2	Metabolic syndrome	41.5444	0.00078	−0.013	0.172
	Waist circumference	45.709	0.00054	−0.001	0.655
	Essential hypertension	52.127	3.577239e−05	0.00038	0.710
	FBG	165.927	1.777002e−25	0.0036	0.476
	HDL cholesterol	11.660	0.040	0.0060	0.738
	Triglycerides	158.825	1.761767e−32	0.001	0.988

MMP9	Metabolic syndrome	26.749	0.084	−0.002	0.777
	Waist circumference	37.119	0.007667765	0.003	0.164
	Essential hypertension	17.634	0.48	−0.0001	0.795
	FBG	18.492	0.490	0.002	0.174
	HDL cholesterol	7.909	0.161	−0.005	0.462
	Triglycerides	4.963	0.420	−0.003	0.636

MMP16	Metabolic syndrome	23.772	0.022	−0.001	0.939
	Waist circumference	11.474	0.245	0.001	0.841
	Essential hypertension	15.120	0.235	−0.001	0.165
	FBG	8.535	0.742	0.002	0.345
	HDL cholesterol	6.575	0.160	−0.005	0.746
	Triglycerides	3.495	0.479	0.011	0.340

Abbreviations: FBG, fasting blood glucose; HDL, high‐density lipoprotein; IVW, inverse variance weighted.

**TABLE 3:** MR‐PRESSO test.

Exposure	Outcome	Raw		Outlier corrected		Global P	Number of outliers	Distortion P
		OR (CI%)	*p*	OR (CI%)	*p*			
MMP3	Metabolic syndrome	0.9831 (0.9385–1.0299)	0.483	0.983 (0.9511–1.016)	0.325	0.003	2	0.990
	Waist circumference	0.9934 (0.9824–1.0045)	0.258	0.9967 (0.9892–1.0042)	0.402	0.017	2	0.090
	Essential hypertension	0.9973 (0.9951–0.9996)	0.030	NA (NA ‐ NA)	NA	0.870	0	NA
	FBG	1.0046 (0.9942–1.0151)	0.395	NA (NA ‐ NA)	NA	0.010	0	NA
	HDL cholesterol	(0.9719–1.1081)	0.319	NA (NA ‐ NA)	NA	0.387	0	NA
	Triglycerides	0.9541 (0.8752–1.0402)	0.335	NA (NA ‐ NA)	NA	0.250	0	NA

MMP	Metabolic syndrome	1.0021 (0.9859–1.0187)	0.810	NA (NA ‐ NA)	NA	0.727	0	NA
	Waist circumference	0.9988 (0.9921–1.0055)	0.738	NA (NA ‐ NA)	NA	0.383	0	NA
	Essential hypertension	0.9995 (0.9988–1.0002)	0.244	NA (NA ‐ NA)	NA	0.990	0	NA
	FBG	0.9968 (0.9899–1.0037)	0.414	NA (NA ‐ NA)	NA	0.308	0	NA
	HDL cholesterol	NA (NA ‐ NA)	NA	NA (NA ‐ NA)	NA	NA	NA	NA
	Triglycerides	NA (NA ‐ NA)	NA	NA (NA ‐ NA)	NA	NA	NA	NA

MMP1	Metabolic syndrome	1.0128 (0.9818–1.0448)	0.4319224	NA (NA ‐ NA)	NA	0.140	0	NA
	Waist circumference	1.002 (0.9914–1.0126)	0.7162616	1.0025 (0.9938–1.0113)	0.5732768	< 0.003	2	0.893
	Essential hypertension	1.0006 (0.9972–1.004)	0.717	NA (NA ‐ NA)	NA	0.073	0	NA
	FBG	0.9954 (0.9893–1.0015)	0.153	NA (NA ‐ NA)	NA	0.900	0	NA
	HDL cholesterol	NA (NA ‐ NA)	NA	NA (NA ‐ NA)	NA	NA	NA	NA
	Triglycerides	NA (NA ‐ NA)	NA	NA (NA ‐ NA)	NA	NA	NA	NA

MMP10	Metabolic syndrome	0.9752 (0.9431–1.0084)	0.157	NA (NA ‐ NA)	NA	0.057	0	NA
	Waist circumference	1.0009 (0.9829–1.0193)	0.920	0.997 (0.9899–1.0041)	0.412	< 0.003	1	0.330
	Essential hypertension	1.0024 (0.9981–1.0067)	0.291	1.0007 (0.9972–1.0041)	0.712	< 0.001	1	0.029
	FBG	1.0066 (0.9916–1.0219)	0.398	1.0013 (0.9908–1.0119)	0.818	< 0.001	2	0.101
	HDL cholesterol	0.9678 (0.9242–1.0133)	0.212	NA (NA ‐ NA)	NA	0.287	0	NA
	Triglycerides	0.9597 (0.8889–1.0362)	0.333	0.9258 (0.8359–1.0255)	0.214	0.043	2	0.434

MMP7	Metabolic syndrome	1.0065 (0.965–1.0498)	0.766	NA (NA ‐ NA)	NA	0.077	0	NA
	Waist circumference	1.006 (0.991–1.0212)	0.447	1.009 (1.0004–1.0177)	0.054	< 0.001	2	0.546
	Essential hypertension	0.9997 (0.995–1.0044)	0.889	0.9988 (0.9947–1.0029)	0.577	0.038	1	0.722
	FBG	1.0052 (0.9954–1.0151)	0.312	NA (NA ‐ NA)	NA	0.574	0	NA
	HDL cholesterol	0.9972 (0.9721–1.0229)	0.835	NA (NA ‐ NA)	NA	0.858	0	NA
	Triglycerides	0.963 (0.9305–0.9966)	0.075	NA (NA ‐ NA)	NA	0.559	0	NA

MMP12	Metabolic syndrome	1.0066 (0.9779–1.0362)	0.659	NA (NA ‐ NA)	NA	0.253	0	NA
	Waist circumference	0.9984 (0.9886–1.0083)	0.753	NA (NA ‐ NA)	NA	0.017	0	NA
	Essential hypertension	0.9958 (0.9939–0.9978)	0.0003	NA (NA ‐ NA)	NA	0.953	0	NA
	FBG	0.9927 (0.9866–0.9988)	0.027	NA (NA ‐ NA)	NA	0.666	0	NA
	HDL cholesterol	0.9889 (0.9645–1.0139)	0.415	NA (NA ‐ NA)	NA	0.477	0	NA
	Triglycerides	1.0225 (0.9954–1.0503)	0.155	NA (NA ‐ NA)	NA	0.420	0	NA

MMP2	Metabolic syndrome	0.981 (0.9297–1.0351)	0.4937102	1.0031 (0.9646–1.0432)	0.8782514	0.001	1	0.061
	Waist circumference	1.01 (0.99–1.02)	0.4682030	1 (0.99–1.01)	0.5144882	< 0.001	2	0.299
	Essential hypertension	1 (0.99–1)	0.7209101	1 (0.99–1)	0.8836653	< 0.001	2	0.196
	FBG	1.01 (0.98–1.04)	0.3667337	1 (0.99–1.01)	0.4206551	< 0.001	1	< 0.001
	HDL cholesterol	1.04 (0.97–1.11)	0.3391943	NA (NA ‐ NA)	NA	0.055	NA	NA
	Triglycerides	0.84 (0.66–1.07)	0.2102306	0.95 (0.91–0.99)	0.1254097	< 0.001	3	< 0.001

MMP9	Metabolic syndrome	1.0081 (0.9723–1.0453)	0.6667267	NA (NA ‐ NA)	NA	0.1	NA	NA
	Waist circumference	1 (0.99–1.01)	0.8100796	1 (1–1.01)	0.2785536	0.013	1	0.66
	Essential hypertension	1 (1–1)	0.2399375	NA (NA ‐ NA)	NA	0.557	NA	NA
	FBG	1 (1–1.01)	0.3006407	NA (NA ‐ NA)	NA	0.515	NA	NA
	HDL cholesterol	0.98 (0.94–1.02)	0.4036673	NA (NA ‐ NA)	NA	0.247	NA	NA
	Triglycerides	1.01 (0.98–1.04)	0.4981523	NA (NA ‐ NA)	NA	0.55	NA	NA

MMP16	Metabolic syndrome	0.9973 (0.9557–1.0408)	0.904	NA (NA ‐ NA)	NA	0.017	0	NA
	Waist circumference	1.0046 (0.9947–1.0146)	0.380	NA (NA ‐ NA)	NA	0.234	0	NA
	Essential hypertension	1.0002 (0.9967–1.0037)	0.912	NA (NA ‐ NA)	NA	0.230	0	NA
	FBG	1.0022 (0.9942–1.0101)	0.604	NA (NA ‐ NA)	NA	0.761	0	NA
	Waist circumference	0.9967 (0.9867–1.0068)	0.533	NA (NA ‐ NA)	NA	0.115	0	NA
	HDL cholesterol	0.9983 (0.9577–1.0406)	0.939	NA (NA ‐ NA)	NA	0.236	0	NA
	Triglycerides	0.9793 (0.9516–1.0078)	0.212	NA (NA ‐ NA)	NA	0.652	0	NA

Abbreviations: CI, confidence interval; FBG, fasting blood glucose; HDL, high‐density lipoprotein; NA, not available; OR, odds ratio.

**FIGURE 3:**
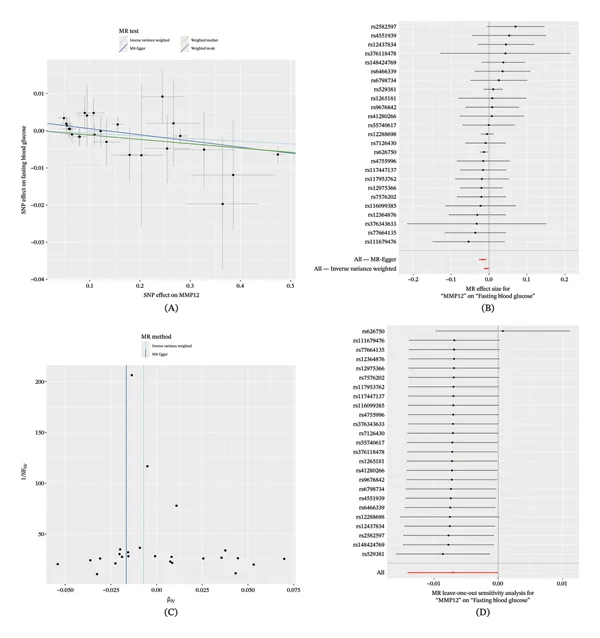
MR analysis and sensitivity analysis of causal relationship between MMP12 and FBG. (A) Scatter plot of the association between MMP12 and FBG. The 4 MR methods applied in this study were all depicted, including IVW, MR‐Egger, weight mode, and weight median MR methods. (B) Forest plot that showed the MR estimate and 95% CI values for each included SNP, as well as the IVW and MR‐Egger results. (C) Funnel plot to detect whether the observed association between MMP12 and FBG was along with obvious heterogeneity. (D) Leave‐one‐out analysis to evaluate whether any single instrumental variable was driving the causal effect of MMP12 on FBG.

In addition, MMP12 may have a suggestive protective effect on FBG levels (IVW: OR = 0.993, 95% CI: 0.986–1, *p* = 0.049; MR‐Egger: OR = 0.984, 95% CI: 0.973–0.994, *p* = 0.005; weighted median: OR = 0.989, 95% CI: 0.98–0.998, *p* = 0.015; weighted mode: OR = 0.988, 95% CI: 0.98–0.996, *p* = 0.009) (Table 2, Figure 3A and B). However, this finding may be affected by potential horizontal pleiotropy and requires further validation.

In the sensitivity analysis, IVW Cochran’s Q test indicated no significant heterogeneity (*p* > 0.05). However, the association between MMP12 and FBG exhibited evidence of horizontal pleiotropy, warranting further validation (MR‐Egger intercept:*p* = 0.022) (Table 2). This finding may partly reflect pleiotropic pathways, whereas the symmetry of the funnel plot indicated negligible biases (Figure 3C), and the LOO analysis further confirmed the robustness of the MR estimates (Figure 3D).

Regarding MetS and its other components, apart from the associations observed for MMP12 with essential hypertension and FBG, the MR results did not provide evidence that other MMPs were causally related to MetS, waist circumference, HDL cholesterol, or triglycerides, with ORs close to 1 and *p*‐values greater than 0.05. In summary, our findings suggested a causal association between MMP12 and essential hypertension. In contrast, the MR analyses demonstrated no causal effects of other MMPs on MetS or its components.

## 4. Discussion

This study employed MR approach to systematically evaluate the causal associations between nine MMPs and MetS and its components. By incorporating 178 IVs, we found that genetically predicted MMP12 levels were robustly inversely associated with the risk of essential hypertension. This result was consistent across multiple MR methods. Additionally, MMP12 showed a suggestive inverse association with FBG; however, this association was accompanied by evidence of horizontal pleiotropy and should therefore be interpreted with caution. No clear causal associations were identified between the remaining MMPs and MetS or its individual components (waist circumference, HDL cholesterol, and triglycerides). These findings suggest that MMP12 may specifically participate in the regulation of blood pressure, rather than exerting a direct causal effect on MetS as a whole.

The protective association between genetically predicted MMP12 and essential hypertension suggests a context‐dependent role of MMP12 in vascular ECM remodeling rather than a simple pro‐inflammatory effect. Pathological vascular remodeling, arterial stiffening, and reduced distensibility are established links between ECM dysregulation and hypertension, and recent MMP12‐focused vascular disease literature further emphasizes context‐dependent mechanisms in arterial remodeling [40, 41]. Previous genetic and experimental evidence also connects MMP12 with blood pressure regulation, including reported associations between MMP polymorphisms and essential hypertension [42], observational links between MMP12 and vascular inflammation [43], and experimental evidence that MMP12 deletion may attenuate age‐related arterial stiffening [44]. Taken together, MMP12 may influence vascular structure and inflammatory signaling in a context‐dependent manner, but it should be interpreted as a candidate pathway for future hypertension risk stratification research after external validation rather than as an established clinical marker.

The association between MMP12 and FBG should be interpreted more cautiously. Although the IVW estimate reached nominal significance (*p* = 0.049), the result was close to the statistical boundary and was accompanied by evidence of horizontal pleiotropy (MR‐Egger intercept *p* = 0.022). Experimental studies link MMP12 to glucose metabolism, intestinal homeostasis, macrophage activity, and inflammatory signaling [45–47], while newer evidence indicates that intestinal immune signaling can regulate systemic lipid and glucose metabolism [48]; together, these findings provide biologically plausible mechanisms but do not establish clinical utility. The observed association may partly reflect shared inflammatory, adipose‐tissue remodeling, intestinal homeostasis, immune, or unmeasured confounding pathways; therefore, the present data do not support using MMP12 as a diagnostic indicator for dysglycemia or MetS.

The absence of a clear causal association between MMP12 and composite MetS may reflect the heterogeneity of MetS itself. MetS and CKM literature emphasizes that this condition integrates several partially distinct metabolic, renal, and vascular phenotypes rather than a single biological pathway [1, 49–51]. MMP‐related ECM remodeling and low‐grade metabolic inflammation may be more relevant to specific components, particularly blood pressure and glucose traits, than to the composite MetS endpoint. This component‐specific pattern also explains why MMP12 may show signals for essential hypertension and FBG while not demonstrating a direct causal effect on MetS as a whole.

This study has several strengths, including the MR design, the use of multiple complementary estimators, sensitivity analyses, and large‐scale GWAS datasets for MetS‐related traits. This study has several strengths, including the MR design, the use of multiple complementary estimators, comprehensive sensitivity analyses, and large‐scale GWAS datasets for MetS‐related traits. Nevertheless, several limitations should be acknowledged. First, the majority of the outcome datasets were derived from populations of European ancestry, which may limit the generalizability of our findings to other ethnic groups. Second, due to the limited number of SNP available for certain MMPs, we employed a relatively liberal IV selection threshold (*p* < 5 × 10^−6^). Although all selected instruments demonstrated F‐statistics greater than 10, the potential for weak instrument bias and unmeasured pleiotropic variants cannot be entirely excluded. Third, the circulating plasma protein levels used as exposures in this MR analysis may not fully reflect the local tissue‐specific expression and activity of MMPs (e.g., within the vascular wall or adipose tissue) that are critical for cardiometabolic regulation. Fourth, standard two‐sample MR assumes a linear relationship between exposure and outcome; thus, potential nonlinear effects of MMP12 could not be evaluated. Finally, while MR provides genetic evidence for causal inference, it cannot replace experimental functional validation. The suggestive association between MMP12 and FBG remains vulnerable to horizontal pleiotropy and requires further rigorous mechanistic investigation.

Future research should validate these findings in multi‐ancestry cohorts, incorporate sex‐specific and biosample‐specific protein analyses, perform bidirectional MR where appropriate, and use functional experiments to clarify the causal pathways suggested by the genetic evidence. If replicated, MMP12‐related pathways may support future biomarker research, risk stratification, or therapeutic‐target exploration, but the present findings should be regarded as hypothesis‐generating rather than immediately clinically actionable. Recent obesity‐intervention, MMP‐activity, and CKM health literature provides appropriate background for this validation agenda, but should be used only to frame future research rather than to infer that MMP12 diagnoses MetS [50–53].

## 5. Conclusions

In conclusion, this MR study provides genetic evidence that MMP12 may be associated with specific cardiometabolic components, especially essential hypertension, while its association with FBG remains suggestive and potentially affected by horizontal pleiotropy. The findings do not support MMP12 as a diagnostic biomarker for composite MetS, but they highlight MMP12‐related pathways as candidates for future validation and mechanistic research.

## Author Contributions

All authors contributed to the study conception and design. Xinming Li and Zeguo Shao designed the research, participated in acquisition, analysis, or interpretation of data, and drafted the manuscript. Qiaohui Qian and Ming Fang performed the statistical analysis, participated in its design, and drafted the manuscript. Zhaohua Gu, Yaming Yan, He Wen, and Qian Sheng carried out the studies and participated in collecting data.

## Funding

This work was supported by the Investigator‐Initiated Trial project provided by Shanghai Pudong New Area Health Commission (No. 2025‐PWDL‐19) and the Special Fund Project of Scientific Research on Community Medicine and Health Management provided by Shanghai Association of Integrated Traditional Chinese and Western Medicine (No. 2023SQ14).

## Disclosure

All authors read and approved the final manuscript.

## Ethics Statement

All GWAS data from the included articles were approved by corresponding ethics committees, and participants provided written informed consent.

## Consent

The authors have nothing to report.

## Conflicts of Interest

The authors declare no conflicts of interest.

## Supporting Information

Additional supporting information can be found online in the Supporting Information section.

## Supporting information


**Supporting Information** Supporting Table 1 Basic GWAS information in each exposure/outcome‐related trait. This table provides an overview of the GWAS datasets utilized in the two‐sample MR analyses. For each exposure and outcome, it lists the trait type (exposure or outcome), the GWAS ID number, the sample size (reported as the number of cases and controls where applicable), and the total number of SNPs included in the respective GWAS. The exposures consist of MMPs (MMP, MMP1, MMP2, MMP3, MMP7, MMP9, MMP10, MMP12, and MMP14), and the outcomes include metabolic syndrome, waist circumference, essential hypertension, FBG, HDL cholesterol, and triglycerides. Further details on the study populations and data sources are provided in the Methods section. Supporting Table 2 Summary of basic instrument variables information. This table provides the harmonized single‐nucleotide polymorphism (SNP)‐level data used as IVs for each exposure‐outcome pair. For every SNP included in the MR analyses, the table reports the effect allele and other allele for both exposure and outcome, beta coefficients and standard errors, effect allele frequencies, *p*‐values, chromosome and position, and the F‐statistic of the SNP. It also indicates whether a SNP was retained (mr_keep) after quality control, including filters for palindromic variants and linkage disequilibrium clumping. Supporting Table 3 F statistics of the included instrument variables in each MR analysis. This table presents the summary statistics of instrument strength for each exposure across all tested outcomes. For MMP3, MMP, MMP1, MMP10, MMP7, MMP12, MMP2, MMP9, and MMP16, it reports the number of SNPs used, and the mean, minimum, and maximum F‐statistic. All mean F‐statistics exceed the conventional threshold of 10, indicating that weak instrument bias is unlikely to affect the results. STROBE‐MR‐checklist. STROBE‐MR checklist of recommended items to address in reports of Mendelian randomization studies

## Data Availability

All data generated or analyzed during this study are included in this published article and its Supporting Information files.
