# Supplementary material for: Evaluating the Causal Roles of Matrix Metalloproteinases in Metabolic Syndrome: A Mendelian Randomization Study Highlighting MMP12 in Blood Pressure Regulation
Source: Genet Res (Camb). 2026 Aug 30;2026:1665162. doi: 10.1155/genr/1665162 (PMC13527163; doi:10.1155/genr/1665162)
Supplement: Supplementary file 1 [file GENR-2026-1665162-s001.zip › Supplementary tables.docx]

**Supplementary material**

**Supplementary Table 1 Basic GWAS information in each exposure/outcome-related trait.**

| Trait | Character | GWAS ID | N. case/control | N. SNPs | Population | PMID |
| --- | --- | --- | --- | --- | --- | --- |
| MMP | exposure | ebi-a-GCST90000487 | 982 | 7,883,813 | European | 3264108 |
| MMP3 | exposure | ebi-a-GCST90012027 | 21,758 | 12,705,782 | European | 33067605 |
| MMP1 | exposure | ebi-a-GCST90012033 | 21,758 | 12,705,782 | European | 33067605 |
| MMP10 | exposure | ebi-a-GCST90012050 | 21,758 | 12,705,782 | European | 33067605 |
| MMP7 | exposure | ebi-a-GCST90012056 | 21,758 | 12,705,782 | European | 33067605 |
| MMP12 | exposure | ebi-a-GCST90012070 | 21,758 | 12,705,782 | European | 33067605 |
| MMP2 | exposure | GCST90088616 | 5,364 | 7,506,463 | European | 35078996 |
| MMP9 | exposure | GCST90087966 | 5,357 | 7,506,463 | European | 35078996 |
| MMP14 | exposure | GCST90241890 | 3,301 | 10,534,735 | European | 29875488 |
| metabolic syndrome | outcome | GCST009602 | 59,677/231,430 | NA | European | 31589552 |
| Waist circumference | outcome | ebi-a-GCST90014020 | 407,661 | 10,783,687 | European | 34017140 |
| Essential hypertension | outcome | ukb-b-12493 | 54,358/408,652 | 9,851,867 | European | 9851867 |
| FBG | outcome | ebi-a-GCST90002232 | 200,622 | 31,008,728 | European | 34059833 |
| HDL cholesterol | outcome | GCST002223 | 94,595 | NA | European | 24097068 |
| Triglycerides | outcome | GCST002216 | 94,595 | NA | European | 24097068 |

GWAS, genome-wide association studies; N. case/control, number of case/control; N. SNPs, number of SNPs; FBG, Fasting blood glucose; HDL, High-Density Lipoprotein.

**Supplementary Table 3. F statistics of the included instrument variables in each MR analysis.**

| Exposure | SNP | Mean F | Min F | Max F |
| --- | --- | --- | --- | --- |
| MMP3 | 21 | 127.3438 | 20.88584 | 1464.149 |
| MMP | 5 | 30.83749 | 20.83924 | 65.46224 |
| MMP1 | 25 | 74.0639 | 20.8279 | 1037.209 |
| MMP10 | 24 | 71.0924 | 21.25251 | 647.8325 |
| MMP7 | 23 | 48.34235 | 20.92565 | 523.6863 |
| MMP12 | 26 | 99.96208 | 20.71335 | 1395.218 |
| MMP2 | 20 | 23.14764 | 20.92182 | 28.30871 |
| MMP9 | 21 | 28.68019 | 21.14551 | 120.59045 |
| MMP16 | 13 | 22.39092 | 21.03059 | 28.84871 |
